# Comparison of O-RADS with the ADNEX model and IOTA SR for risk stratification of adnexal lesions: a systematic review and meta-analysis

**DOI:** 10.3389/fonc.2024.1354837

**Published:** 2024-05-02

**Authors:** Jing Han, Jing Wen, Wei Hu

**Affiliations:** ^1^Department of Radiology, Suzhou Hospital, Affiliated Hospital of Medical School, Nanjing University, Suzhou, China; ^2^Department of Medical Imaging, Jiangsu Vocational College of Medicine, Yancheng, China; ^3^Department of Radiology, Yixing Traditional Chinese Medicine Hospital, Yixing, China

**Keywords:** O-RADS, IOTA, ADNEX model, ovarian cancer, diagnostic

## Abstract

**Purpose:**

This study aims to systematically compare the diagnostic performance of the Ovarian-Adnexal Reporting and Data System with the International Ovarian Tumor Analysis Simple Rules and the Assessment of Different NEoplasias in the adneXa model for risk stratification of ovarian cancer and adnexal masses.

**Methods:**

A literature search of online databases for relevant studies up to July 2023 was conducted by two independent reviewers. The summary estimates were pooled with the hierarchical summary receiver-operating characteristic model. The quality of the included studies was assessed with the Quality Assessment of Diagnostic Accuracy Studies–2 and the Quality Assessment of Diagnostic Accuracy Studies-Comparative Tool. Metaregression and subgroup analyses were performed to explore the impact of varying clinical settings.

**Results:**

A total of 13 studies met the inclusion criteria. The pooled sensitivity and specificity for eight head-to-head studies between the Ovarian-Adnexal Reporting and Data System and the Assessment of Different NEoplasias in the adneXa model were 0.96 (95% CI 0.92–0.98) and 0.82 (95% CI 0.71–0.90) vs. 0.94 (95% CI 0.91–0.95) and 0.83 (95% CI 0.77–0.88), respectively, and for seven head-to-head studies between the Ovarian-Adnexal Reporting and Data System and the International Ovarian Tumor Analysis Simple Rules, the pooled sensitivity and specificity were 0.95 (95% CI 0.93–0.97) and 0.75 (95% CI 0.62–0.85) vs. 0.91 (95% CI 0.82–0.96) and 0.86 (95% CI 0.76–0.93), respectively. No significant differences were found between the Ovarian-Adnexal Reporting and Data System and the Assessment of Different NEoplasias in the adneXa model as well as the International Ovarian Tumor Analysis Simple Rules in terms of sensitivity (*P* = 0.57 and *P* = 0.21) and specificity (*P* = 0.87 and *P* = 0.12). Substantial heterogeneity was observed among the studies for all three guidelines.

**Conclusion:**

All three guidelines demonstrated high diagnostic performance, and no significant differences in terms of sensitivity or specificity were observed between the three guidelines.

## Introduction

Ovarian carcinoma is the leading cause of mortality from gynecological malignancy in the USA, where approximately more than 13,000 deaths are from ovarian carcinoma in 2023 and the 5-year survival rate is no more than 50% (1). The early diagnosis of ovarian carcinoma is associated with a significantly higher 5­year survival rate, which is increased to >90% for stage 1 (2). Therefore, it is important to accurately differentiate malignant tumors from benign tumors, thereby optimizing patient triaging and reducing unnecessary surgeries without missing cancer. Although several imaging modalities such as MRI and CT play a role in the assessment and management of adnexal lesions, ultrasound (US) is still the first-line preoperative differential diagnosis method for ovarian masses (3, 4).

Several risk stratification systems have been developed to standardize the assessment of adnexal masses with US to improve accuracy and interreader agreement. The International Ovarian Tumor Analysis (IOTA) group proposed terminology and definitions to describe ultrasound features of adnexal lesions in 2008, aiming to provide a standardized tool for differentiating benign and malignant adnexal lesions (5, 6). The IOTA Simple Rules (IOTA SR) includes five descriptions for benignity (benign features) and five for malignancy (malignant features), and adnexal masses are classified as benign, malignant, and inconclusive. Previous studies showed that the IOTA SR has high performance, with a pooled sensitivity of 0.93 and specificity of 0.80 (7). However, the IOTA SR is unable to classify all adnexal masses, leaving as much as 25% inconclusive lesions; when both malignant and benign features were present, or if none of the features were present, the simple rules were inconclusive (6).

In 2014, the IOTA group developed a new scoring system named the Assessment of Different NEoplasias in the adneXa (ADNEX) model, which used three clinical variables and six ultrasound variables to calculate the risk of an adnexal lesion (benign or malignant), distinguishing four types of malignant ovarian tumors: borderline, stage I cancer, stage II–IV cancer, and secondary metastatic cancer (8–10). Additionally, other standardized guidelines or risk stratification systems were proposed such as the Gynecologic Imaging Reporting and Data System (GI-RADS), the Risk of Malignancy Index 4 (RMI4), and the logistic regression model 2 (LR2) (11–13). Nonetheless, many of these standardized models were found inferior to subjective expert assessment (14). In 2018, based on IOTA terms and data sets, the American College of Radiology (ACR) introduced the Ovarian-Adnexal Reporting and Data System (O-RADS) US risk stratification and management (15). With O-RADS US, an adnexal mass is stratified to a 1–5 category (1, physiologic; 2, almost certainly benign; 3, low risk of malignancy; 4, intermediate of malignancy; 5, high risk of malignancy) according to its sonographic features. Since the publication of O-RADS US, a number of studies evaluating this scoring system have been published. Additionally, some of them had performed head-to-head comparisons between O-RADS US with other guidelines. Although several meta-analyses or systematic reviews have summarized the diagnostic accuracy of O-RADS US, a comparison with other guidelines has not been reported systematically. Therefore, in this study, we aimed to systematically compare the performance of O-RADS US with IOTA SR and the ADNEX model.

## Materials and methods

This meta-analysis and systematic review adhered to the Preferred Reporting Items for Systematic Reviews and Meta-Analysis (PRISMA) statement (16). The primary outcome of this study was the direct comparison between O-RADS and IOTA SR along with the ADNEX model. Furthermore, the overall diagnostic performance of O-RADS for all the included studies was calculated.

### Search strategy and selection criteria

An electronic search of PubMed, EMBASE, Cochrane Library, Web of Science, and Google Scholar online scientific publication databases was conducted to identify relevant studies that were published up to 31 July 2023, with language restricted to English only. The following terms in combination with abbreviations were used for the literature search: (“O-RADS” OR “Ovarian-Adnexal Reporting and Data System”) AND [(“IOTA SR” OR “SR” OR “simple rules” OR “International Ovarian Tumor Analysis SR” OR “IOTA simple rules”) OR (“ADNEX” OR “ADNEX models” OR “IOTA ADNEX”)]. An additional literature search was supplemented by manually screening the bibliographies among the included studies and reviews to prohibit missing potential eligible studies. Two reviewers (H.J. and W.J.) independently assessed the search results, and any disagreements were resolved through discussion until a consensus was reached.

### Inclusion and exclusion criteria

Studies that met all of the following criteria were included: 1) used O-RADS and IOTA SR and/or the ADNEX model for the risk stratification of adnexal lesions, with head-to-head comparisons of diagnostic accuracy; 2) provided sufficient details to construct 2 × 2 contingency tables for determining diagnostic accuracy; and 3) had surgical pathology results or at least 1-year follow-up as the reference standard. Studies that met any of the following criteria were excluded: 1) had no direct comparison between O-RADS with the other guidelines; 2) did not report sufficient data to assess the diagnostic performance; and 3) were meta-analyses, guidelines, editorials, reviews, conference abstracts, and letters.

### Data extraction and quality assessment

A predefined standardized form was employed to extract the following data from the included studies: 1) clinical and demographic characteristics, e.g., number of patients and lesions, patient age, and tumor size; and 2) study characteristics, e.g., first author, study design (prospective or retrospective), publication year, location of the study and period, number and experience of radiologists, cutoff values, guidelines, and the reference standard. We used the Quality Assessment of Diagnostic Accuracy Studies–2 (QUADAS-2) to perform quality assessment of the included studies (17), with each study categorized as having either low, unclear, or high risk of bias according to the following four domains: patient selection, method of the index test, reference standard, and flow and timing. The quality of the studies that included a head-to-head comparison of O-RADS US with either the IOTA SR or the ADNEX model was assessed with the QUADAS-Comparative (QUADAS-C), an extension of QUADAS-2 designed for comparative diagnostic performance studies. Two reviewers (H.J. and W.J.) independently conducted the data extraction and quality assessment, with discrepancies resolved through a discussion with a third reviewer (H.W.).

### Data synthesis and statistical analysis

In this meta-analysis, we used the hierarchical summary receiver-operating characteristic (HSROC) model to summarize the estimates of sensitivity, specificity, and their 95% confidence intervals (CIs) (18). Forest plots and HSROC curves were used to graphically present the results. For studies that provided at least two results, we chose the most accurate; for studies that provided validation results of internal validation and external validation, we chose the latter. The Cochran *Q* statistics and Higgins *I*^2^ value were employed to measure the degree of heterogeneity among the studies: *I*^2^ value between 0% and 40%, not important; *I*^2^ value between 30% and 60%, moderate; *I*^2^ value between 50% and 90%, substantial; and *I*^2^ value between 75% and 100%, considerable (19). To explore the source of heterogeneity, the following covariates were used to perform metaregressions: the country where the study was conducted, publication year, number of patients, number of malignancies, and malignant rate. The Deeks’ funnel plot was used to assess the publication bias, and the statistical significance was tested with the Deeks’ funnel plot asymmetry test. All analyses were performed with STATA (version 15.1) and R statistical software (version 3.6.1), with a *P*-value <0.05 indicating statistical significance.

## Results

### Literature search and data extraction

**Figure 1:**
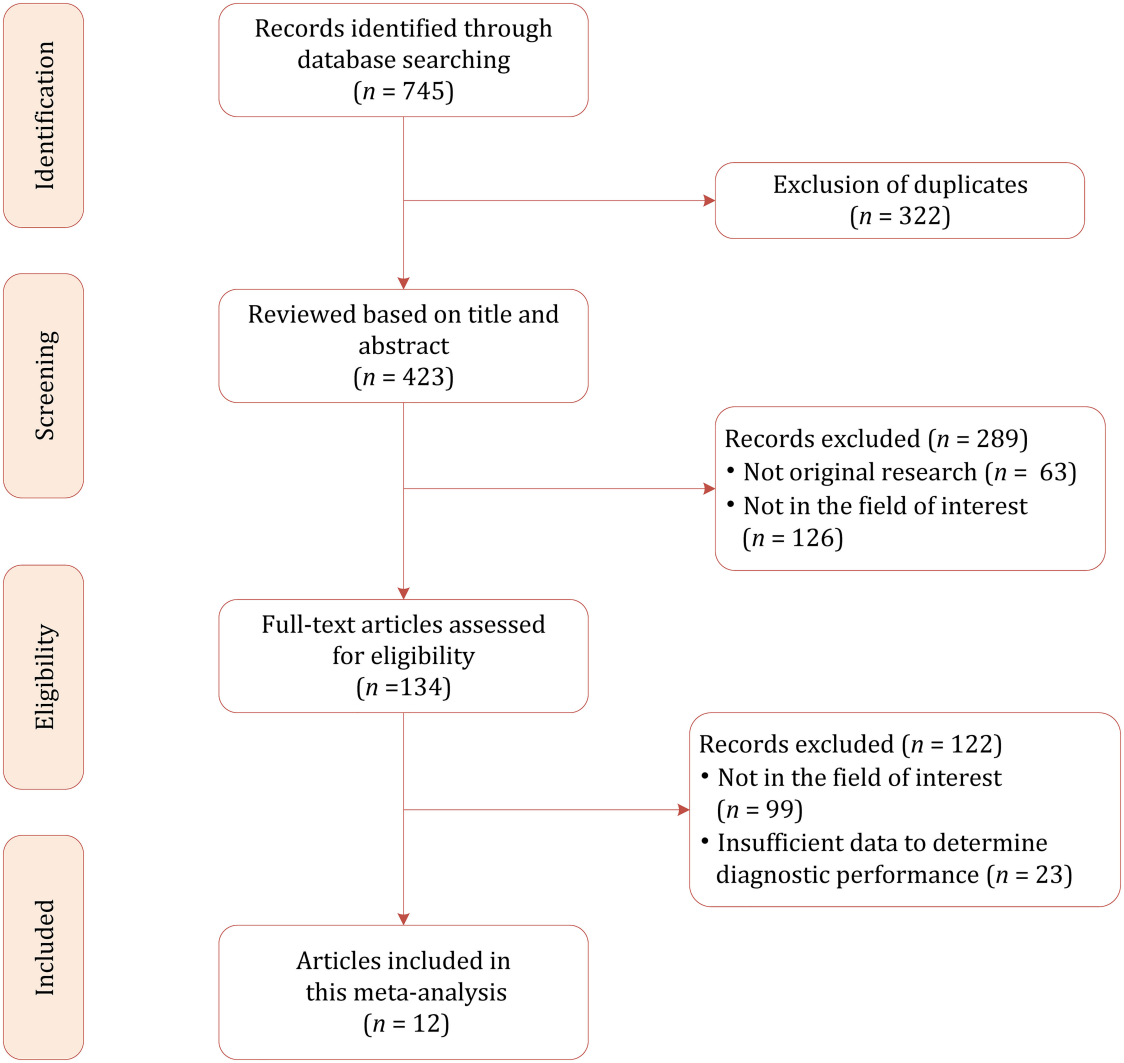
Study selection process for this systematic review and meta-analysis.

Based on our literature search strategy, a total of 745 references were identified initially, of which 322 were excluded for duplicates. After examining the titles and abstracts, 289 results were excluded because they were not relevant to this meta-analysis. We reviewed the remaining 134 full-text articles, and 122 were excluded for reasons as follows: insufficient data to determine diagnostic performance (*n* = 23) and not in the field of interest (*n* = 99). Finally, a total of 12 studies were included in this meta-analysis (20–31). The flowchart of the literature selection process is demonstrated in **Figure 1**.

### Characteristics of the included studies

For this meta-analysis, all the studies included had a retrospective study design, with eight studies reporting a head-to-head comparison between O-RADS and the ADNEX model (21, 23–28, 31) and seven studies reporting a direct comparison between O-RADS and IOTA SR (including three studies performing a comparison between all three guidelines) (20, 22, 23, 25, 29–31). The study sample ranged from 122 to 1,179 patients, with an average age of 35–52.3. The average size of the adnexal mass lesion in seven studies was 60–190 mm. In most studies, surgical pathology results were used as the reference; however, in two studies, a follow-up of 12–24 months was also used when histopathological results were not available (20, 31). Borderline lesions were reported in 11 studies, and all of these studies classified those masses as malignant. Ten studies reported the experience of radiologists, with most of them having at least 5 years of experience. In two studies, readers had partial knowledge of patients’ clinical information (22, 23). The reported kappa values were substantial to almost perfect for the three guidelines: 0.62–0.93 for O-RADS, 0.85–0.86 for the ADNEX model, and 0.73–0.90 for IOTA SR. The most used cutoff values for O-RADS and the ADNEX model were ≥4 and ≥10%, respectively. For IOTA SR, six of seven studies reported details on indeterminate cases, with a prevalence of 5.4%–24.7%. Of these cases, the malignant rate ranged from 43.1% to 48.6%. Details on demographic characteristics and study characteristics are presented in **Tables 1**, **2**.

**Table 1:** Demographic characteristics of the included studies.

First author	Country	Year	No. of patients	No. of lesions	Malignant	Borderline Tumors	Age (year, mean ± SD/median)	Tumor size (mm, mean ± SD/range)	Reference
Basha et al. (20)	Egypt	2021	609	647	178	32	48 ± 13.7	NA	Pathological or 2-year follow-up
Chen et al. (21)	Taiwan	2022	322	322	264	8	44 (20–83)	NA	Pathological
Guo et al. (22)	China	2022	575	592	145	22	36.6 ± 13.9/46.5 ± 13.9^a^	NA	Pathological
Hiett et al.	USA	2021	150	150	40	12	48.2 ± 1.7/47.5 ± 3.1^a^	83.7 ± 5.2/101.4 ± 7.7^a^	Pathological
Lai et al. (24)	China	2021	734	734	170	69	35 (29–46)/48 (36–55.3)^a^	NA	Pathological
Pelayo et al. (25)	Spain	2023	122	122	41	NA	51.4 ± 15.7	94.2 ± 52.1	Pathological
Poonyakanok et al. (26)	Thailand	2023	357	357	61	11	43 (35–53)	60 (42–83.8) 190 (159–277.5)^a^	Pathological
Spagnol et al. (27)	Italy	2023	514	514	89	25	51 (41–63)	NA	Pathological
Wang et al. (28)	China	2023	445	445	180	31	40.19 ± 15.96/52.34 ± 13.15^a^	95 ± 285/605 ± 492^a^	Pathological
Xie et al. (29)	China	2022	453	453	269	48	48.8 ± 13.4	105.9 ± 64	Pathological
Yang et al. (30)	China	2023	1,179	1,179	213	67	38 (29–50)	69 (51–94)	Pathological
Yoeli-Bik et al. (31)	USA	2023	511	511	81	15	45.4 ± 14.8	NA	Pathological (341) or 1-year follow-up

NA, not available; SD, standard deviation.

a Benign/malignant.

**Table 2:** Study characteristics of the included studies.

First author	Publication year	Study design	Analysis	Period	No. of readers	Experience (years)	Blinded	Guideline	*κ* value	Cutoff value
Basha et al. (20)	2021	Retrospective	Per lesion	2016.5–2019.12	5	15	Yes	O-RADS/IOTA SR	0.77/0.63	≥4/NA
Chen et al. (21)	2022	Retrospective	Per person	2020.1–2020.10	5	≥5	Yes	O-RADS/ADNEX	0.78^a^	≥4/>10%
Guo et al. (22)	2022	Retrospective	Per lesion	2017–2020	4	≥10 (2 readers)/1 (2 readers)	Yes^b^	O-RADS/IOTA SR	0.71/0.77	≥4/NA
Hiett et al.	2021	Retrospective	Per person	2018.3–2021.2	2	25	Yes^b^	O-RADS/ADNEX/IOTA SR	NA	≥4/>10%/NA
Lai et al. (24)	2021	Retrospective	Per person	2017.1–2020.11	2	5	Yes	O-RADS/ADNEX	0.83/0.86	≥4/>10%
Pelayo et al. (25)	2023	Retrospective	Per person	2021.01–2022.12	2	≥15	Yes	O-RADS/ADNEX/IOTA SR	NA	≥4/>10%/NA
Poonyakanok et al. (26)	2023	Retrospective	Per person	2018.05–2019.05	6	5–8	Yes	O-RADS/ADNEX	NA	≥4/>10%
Spagnol et al. (27)	2023	Retrospective	Per person	2018.01–2021.12	2	NA	Yes	O-RADS/ADNEX	NA	≥4/>10%
Wang et al. (28)	2023	Retrospective	Per person	2020.01–2021.12	2	>5	NA	O-RADS/ADNEX	0.87/0.85	≥4/>10%
Xie et al. (29)	2022	Retrospective	Per person	2017.1–2020.9	2	5	Yes	O-RADS/IOTA SR	0.62/0.73	≥4/NA
Yang et al. (30)	2023	Retrospective	Per person	2021.09–2022.02	2	10/NA	Yes	O-RADS/IOTA SR	0.93/0.90	≥4/NA
Yoeli-Bik et al. (31)	2023	Retrospective	Per person	2017.1–2022.10	3	20/40/NA	Yes	O-RADS/ADNEX/IOTA SR	NA	≥4/>10%/NA

ADNEX, Assessment of Different NEoplasia in the adneXa model; NA, not available. O-RADS, Ovarian-Adnexal Reporting and Data System; IOTA SR, Ovarian Tumor Analysis Simple Rules.

a Only for O-RADS.

b Known partial clinical information of the patients.

### Quality assessment

**Figure 2:**
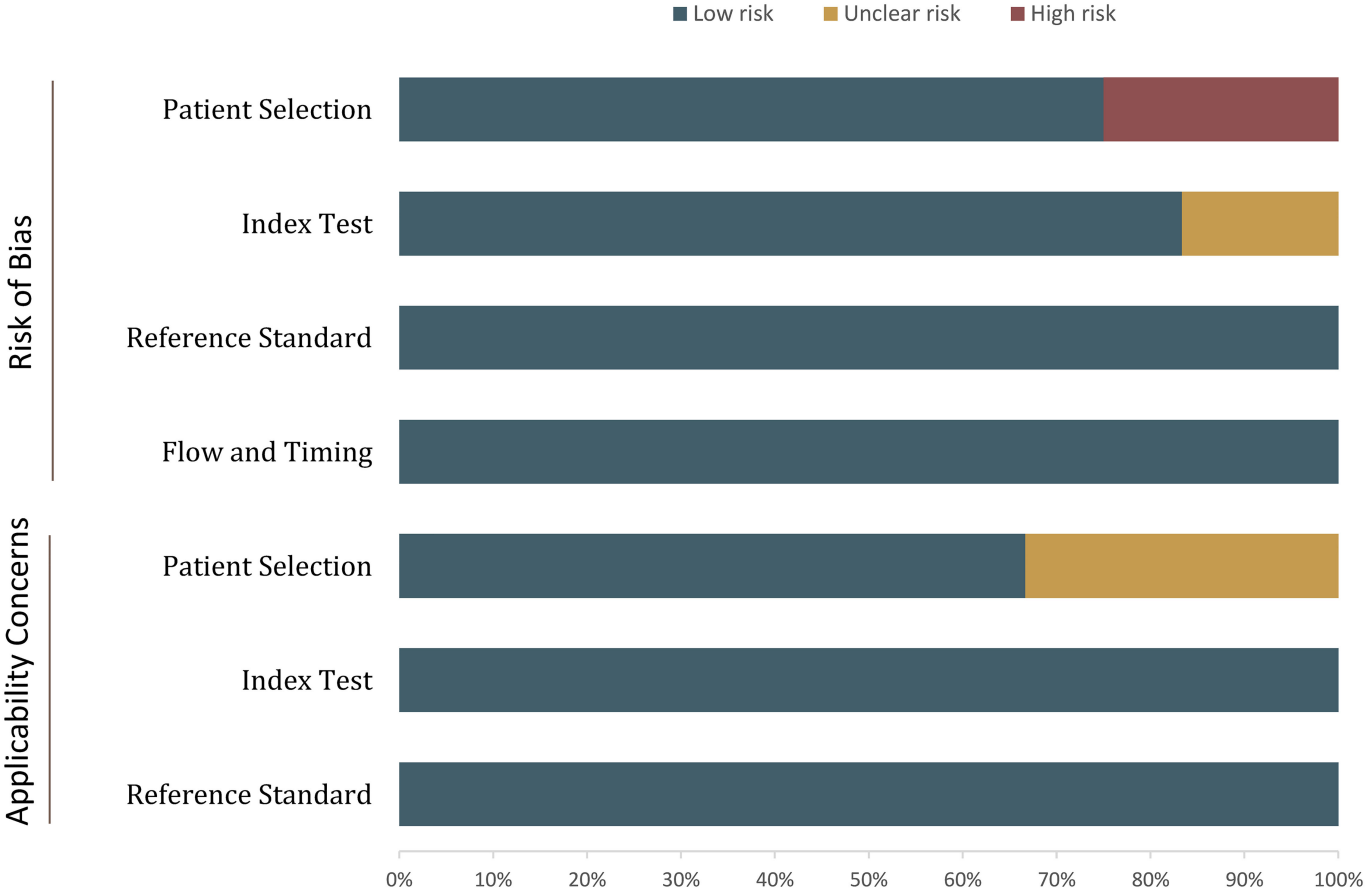
Grouped bar charts show the risk of bias and concerns for applicability of the included studies.

The overall quality assessment using QUADAS-2 is presented in **Figure 2**. For the patient selection domain, five studies had an unclear risk of bias because of two high malignancy rates (20, 23, 25, 28, 29). In three studies, the details on blinding were not provided or reported whether readers have partial knowledge of patient information, thus were assigned an unclear risk of bias in terms of index domain (22, 23, 28). **Supplementary Tables S1, S2** show the details of the quality assessment using QUADAS-2 and QUADAS-C.

### Diagnostic performance

**Figure 3:**
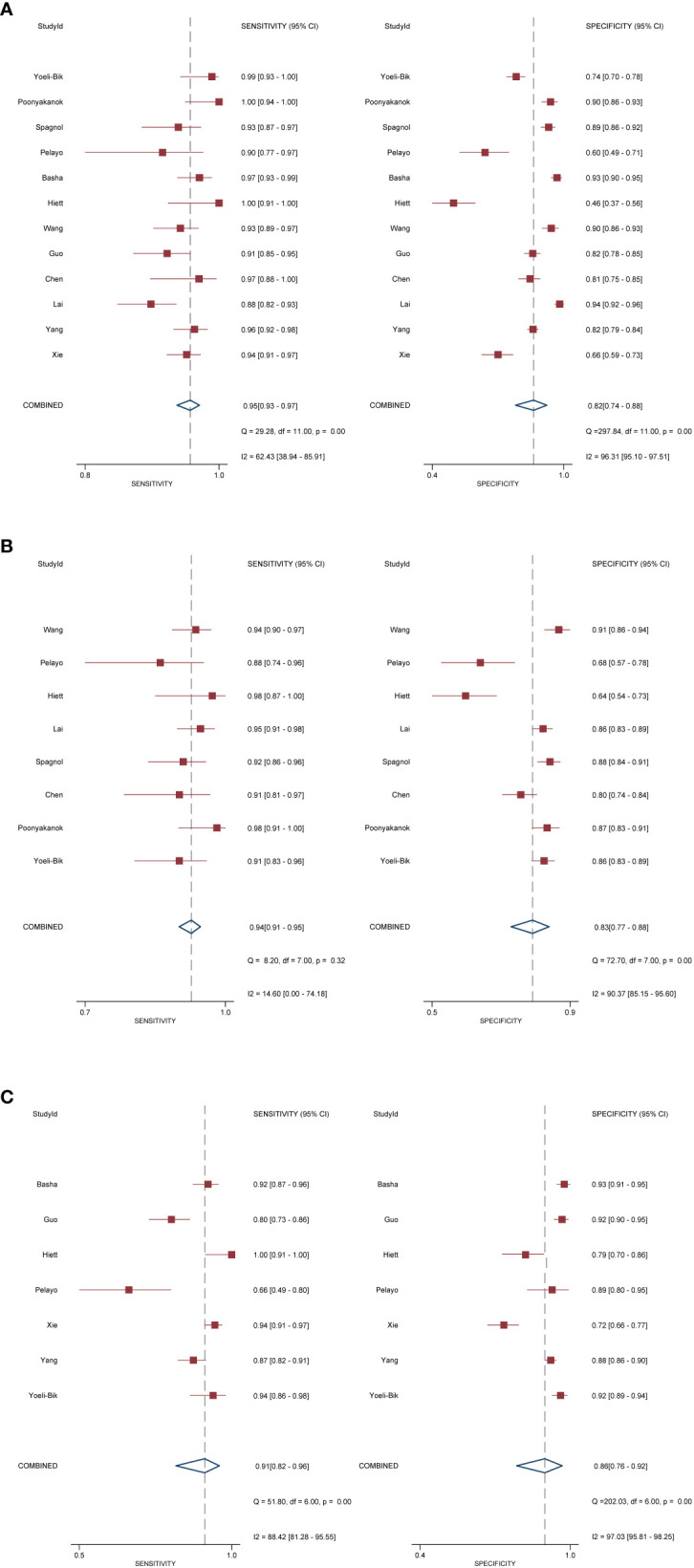
Coupled forest plot of pooled sensitivity and specificity. **(A)** Ovarian-Adnexal Reporting and Data System; **(B)** Assessment of Different NEoplasias in the adneXa; **(C)** International Ovarian Tumor Analysis Simple Rules.

**Figure 4:**
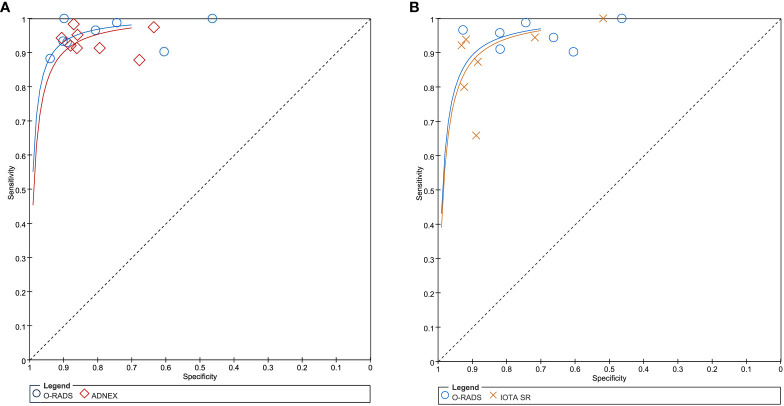
**(A)** Comparison between O-RADS and ADNEX; **(B)** comparison between O-RADS and IOTA SR. ADNEX, Assessment of Different NEoplasias in the adneXa; O-RADS, Ovarian-Adnexal Reporting and Data System; IOTA SR, International Ovarian Tumor Analysis Simple Rules.

In terms of individual studies, the sensitivity and specificity for O-RADS, the ADNEX model, and IOTA SR were 0.88–1.00 and 0.46–0.94, 0.88–0.98 and 0.64–0.91, and 0.80–1.00 and 0.52–0.83, respectively. The pooled sensitivity and specificity for 12 studies using O-RADS at a cutoff value of ≥4 were 0.96 (95% CI 0.92–0.98) and 0.82 (95% CI 0.71–0.90), with an area under the HSROC of 0.97 (95% CI 0.95–0.98); for 8 studies using ADNEX at a cutoff value of ≥10%, the pooled sensitivity and specificity were 0.94 (95% CI 0.91–0.95) and 0.83 (95% CI 0.77–0.88), with an area under the HSROC of 0.95 (0.93–0.96); and for 7 studies using IOTA SR, the pooled sensitivity and specificity were 0.91 (95% CI 0.82–0.96) and 0.86 (95% CI 0.76–0.93), with an area under the HSROC of 0.95 (95% CI 0.92–0.96). The coupled forest plots for O-RADS, the ADNEX model, and IOTA SR are presented in **Figure 3**, and the head-to-head comparisons between the O-RADS and the ADNEX model as well as IOTA SR are presented in **Figure 4**.

Substantial heterogeneity was observed with respect to sensitivity and specificity for all three guidelines. Specifically, the *I*^2^ values for O-RADS were 62.4% (95% CI 38.9%–85.9%) and 96.3% (95% CI 95.1%–97.5%) for sensitivity and specificity; for ADNEX, 14.6% (95% CI 0.0%–74.2%) and 90.4% (95% CI 85.2%–95.6%); and for IOTA SR, 88.4% (95% CI 81.3%–95.6%) and 97.0% (95% CI 95.8%–98.3%), respectively. The space between the 95% confidence region and the 95% prediction region also suggested heterogeneity among the studies (**Supplementary Figure S1**). Metaregression analyses revealed that for O-RADS, the country where the studies were conducted (China vs. other countries) was a significant factor for the heterogeneity of sensitivity (*P* = 0.03), and the number of patients was the significant factor for the ADNEX model (*P* = 0.01) and IOTA SR (*P* = 0.01).

We compared the sensitivity and specificity between guidelines as used in the studies providing direct comparisons. Our analyses demonstrated that no significant differences were found between O-RADS and the ADNEX model, with *P* = 0.57 for sensitivity and *P* = 0.87 for specificity. Likewise, no significant differences were observed between O-RADS and IOTA SR, with *P* = 0.21 for sensitivity and *P* = 0.12 for specificity. The Deeks’ funnel plots demonstrated that there was no publication bias for all three guidelines, with *P*-values of 0.88, 0.22, and 0.87 for O-RADS, the ADNEX model, and IOTA SR.

## Discussion

In this meta-analysis, we systematically compared three guidelines for the risk stratification of ovarian carcinoma. Based on 12 studies, our findings demonstrated that all three risk stratification systems had high diagnostic performance, with the area under the HSROC of 0.97, 0.95, and 0.95 for O-RADS, the ADNEX model, and IOTA SR. No significant differences were found between O-RADS and the ADNEX model (*P* = 0.85) as well as IOTA SR (*P* = 0.15) using the respective eight and seven head-to-head comparison studies. In addition to overall accuracy, we compared the pooled sensitivity and specificity of O-RADS with the ADNEX model and the IOTA SR; however, no significant difference was found between these three guidelines. In the current study, the pooled sensitivity and specificity from 12 studies of O-RADS were 0.95 and 0.82. In two recent meta-analyses evaluating the overall accuracy of O-RADS US, the pooled sensitivity and specificity based on 15 and 10 studies, respectively, were 0.95 and 0.82 and 0.96 and 0.77 at a cutoff value of ≥4, which is comparable with our findings (32, 33). As for IOTA SR and the ADNEX model, the reported pooled sensitivity and specificity from previous meta-analyses or systematic reviews were 0.93 and 0.80 from 5 studies (7) and 0.92 and 0.82 from 10 studies (at the cutoff value of 15%) (34), respectively. In addition to overall diagnostic performance, all three guidelines reported high interreader agreement between radiologists. However, the kappa values were provided only in three studies for IOTA SR and in two studies for the ADNEX model. Therefore, it is unfeasible to perform a meta-analysis and compare the interreader agreement between studies.

The O-RADS US risk stratification and management tool is another effort for the standardization of the risk stratification of adnexal masses, which is modeled on the IOTA rules and based on IOTA data that included 5,905 patients with adnexal masses. Even though IOTA SR had high diagnostic performance, which may result in up to one-quarter of indeterminate lesions, it is suggested that clinicians with less experience need to be assisted by senior clinicians in using diagnostic models to correctly diagnose these lesions (5). In the current study, the reported inconclusive adnexal masses ranged from 5.4% to 24.7%, and nearly half of them were malignant. For unclassified lesions, IOTA SR recommends referring the patient to experts for subjective assessment of US findings, which could provide the most accurate diagnosis. In an earlier study, subjective assessment of adnexal masses using IOTA SR yielded a sensitivity of 89% and a specificity of 80% (5). To address the issue regarding the absence of experienced US examiners, the Simple Rules Risk (SRR) model was developed as a solution, which is a logistic regression model that utilizes TV-US features based on the SR. Its primary objective is to provide an estimated risk of malignancy for any type of adnexal masses, thereby eliminating inconclusive classification. However, because there were only three studies in our meta-analysis that reported the results of SRR, it is unfeasible to pool the data (23, 25, 27).

Compared with IOTA SR, the relatively lower specificity of O-RADS may lead to overtreatment of adnexal masses. However, for the O-RADS indeterminate adnexal masses (categories 3 and 4), the use of O-RADS MRI is suggested for further evaluation of these masses in order to better characterize their nature (32). Compared with the ADNEX model which used three clinical variables and six ultrasound variables, the O-RADS classification only employs ultrasound characteristics to classify ovarian tumors (24, 35). One shortcoming that should be addressed in the present O-RADS is that two variables (bilocular for cystic lesions and shadowing for solid smooth lesions) were not taken into consideration; therefore, it exhibited a higher sensitivity but a lower specificity than the ADNEX model, as reported in various studies (36). Some studies demonstrated that by considering acoustic shadowing as an indication of benign lesions, the overall diagnostic performance was improved significantly, with AUC increased from 0.91 to 0.94 (*P* = 0.01) (37). These findings suggested that acoustic shadowing is an important US feature for classifying ovarian tumors, especially in solid lesions, and should be included. In the updated O-RADS US v2022, the addition of the descriptors bilocular for cystic lesions and acoustic shadowing for solid smooth lesions, along with the expanded lexicon descriptors for the typical appearance of some classic benign lesions, may be beneficial for reducing overtreatment (38).

Although O-RADS, IOTA SR, and the ADNEX model all demonstrated high diagnostic performance, in clinical practice, subjective assessment of pelvic ultrasound images by clinicians with considerable experience in gynecologic ultrasound has demonstrated a high degree of accuracy in differentiating between benign and malignant pelvic lesions (7, 39). In fact, subjective assessment appears to be the best method to predict the likelihood of a pelvic malignancy (40). However, clinicians with this level of expertise may not be universally available, presenting a challenge to accurate diagnosis and patient management. Transferring the expertise of experienced ultrasound examiners to less experienced ones poses a significant challenge in the field of gynecologic US. While scoring systems and risk calculation models can potentially assist less experienced examiners in characterizing pelvic lesions, there are valid criticisms regarding the complexity of US information required by some ultrasound-based risk calculation models, particularly outside of specialist centers. One of the primary criticisms of these models is their reliance on sophisticated ultrasonic features and measurements that may be challenging to obtain consistently and accurately by less experienced examiners. Moreover, the interpretation of ultrasound findings can be subjective and may vary among examiners, leading to potential discrepancies in risk assessment and diagnostic accuracy. Considering the low incidence but high mortality rate, risk stratification of adnexal masses is a trade-off between sensitivity and specificity, which should take into consideration a number of factors such as risk tolerance for missing cancer and surgery risk (31). Therefore, the physician and the patient have to contemplate the risks and benefits of any procedure and determine the individual cutoff in specific circumstances in which the adnexal mass is evaluated.

The main strength of our study is that we systematically summarized currently available evidence on the comparison between O-RADS US with the IOTA SR and the ADNEX model. However, our study has some limitations that must be taken into consideration. First, all studies included in this meta-analysis had a retrospective study design, which was subjected to a selection bias, emphasizing the need for prospective validation. Second, substantial heterogeneity was observed among the studies, which affected the general applicability of our study. To investigate the heterogeneity, we performed metaregression analysis using several potential covariates. Nevertheless, these analyses only accounted for the partial source of heterogeneity, and a portion remains unexplained. Third, comparisons between O-RADS and the ADNEX model as well as the IOTA SR were based on nine and seven studies, respectively; thus, our conclusions and results should be regarded with caution and future large, prospective studies are needed to compare these different guidelines.

## Conclusion

The O-RADS US, the ADNEX model, and IOTA SR showed favorable diagnostic accuracy for risk stratification of adnexal masses, and these three guidelines demonstrated comparable performance. However, O-RADS US yielded a slightly higher sensitivity but a lower specificity than the ADNEX model and IOTA SR.

## Data availability statement

The original contributions presented in the study are included in the article/**Supplementary Material**. Further inquiries can be directed to the corresponding author.

## Author contributions

JH: Data curation, Resources, Writing – original draft. JW: Formal analysis, Methodology, Validation, Writing – review & editing. WH: Supervision, Writing – review & editing.
