# Supplementary material for: Comparison of O-RADS with the ADNEX model and IOTA SR for risk stratification of adnexal lesions: a systematic review and meta-analysis
Source: Front Oncol. 2024 May 2;14:1354837. doi: 10.3389/fonc.2024.1354837 (PMC11096596; doi:10.3389/fonc.2024.1354837)
Supplement: Supplementary file 1 [file Table_1.docx]

**Table S1** Quality assessment according to QUADAS-2

| **Study** | **Risk of bias** | | | | **Concern of applicability** | | | **Reason for high risk of bias** |
| --- | --- | --- | --- | --- | --- | --- | --- | --- |
|  | Patient selection | Index test | Reference standard | Flow and timing | Patient selection | Index test | Reference standard |  |
| Basha et al. | Low | Low | Low | Low | Unclear | Low | Low | Too high malignant rate. |
| Chen et al. | Low | Low | Low | Low | Low | Low | Low |  |
| Guo et al. | High | Low | Low | Low | Low | Low | Low | Patient selection: excluded patients without CA125;  Blinding: not reported explicitly. |
| Hiett et al. | Low | Low | Low | Low | Unclear | Low | Low | Blinding: not reported explicitly;  Too high malignant rate. |
| Lai et al. | Low | Low | Low | Low | Low | Low | Low |  |
| Pelayo et al. | High | Low | Low | Low | Unclear | Low | Low | non-consecutive patients;  Too high malignant rate. |
| Poonyakanok et al. | High | Low | Low | Low | Unclear | Low | Low | details were reported in previous study; |
| Spagnol et al. | Low | Low | Low | Low | Low | Low | Low |  |
| Wang et al. | Low | Unclear | Low | Low | Low | Low | Low | Blinding: not reported explicitly.  Too high malignant rate. |
| Xie et al. | High | Low | Low | Low | Unclear | Low | Low | Patient selection: excluded patients without CA125;  Too high malignant rate. |
| Yang et al. | Low | Unclear | Low | Low | Low | Low | Low |  |
| Yoeli-Bik et al. | Low | Unclear | Low | Low | Low | Low | Low |  |

**Table S2** Quality assessment according to QUADAS-C

| **Study** | **Risk of Bias** | | | | **Reason for high risk of bias** |
| --- | --- | --- | --- | --- | --- |
|  | patient selection | index test | reference standard | flow and timing |  |
| Basha et al. | Low | Low | Low | Low |  |
| Chen et al. | Low | Unclear | Low | Low | Index test: without blinding information |
| Guo et al. | High | Low | Low | Low | patient selection: excluded patients without CA125 |
| Hiett et al. | Low | Low | Low | Low | Index test: without blinding information |
| Lai et al. | Low | Low | Low | Low |  |
| Pelayo et al. | Low | Low | Low | Low |  |
| Poonyakanok et al. | High | Low | Low | Low |  |
| Spagnol et al. | Low | Unclear | Low | Low |  |
| Wang et al. | Low | Low | Low | Low | Index test: without blinding information |
| Xie et al. | High | Low | Low | Low | Patient selection: excluded patients without CA125 |
| Yang et al. | Low | Low | Low | Low |  |
| Yoeli-Bik et al. | Low | Low | Low | Low |  |
