# Supplementary figures and images for: Comparison of O-RADS with the ADNEX model and IOTA SR for risk stratification of adnexal lesions: a systematic review and meta-analysis
Source: Front Oncol. 2024 May 2;14:1354837. doi: 10.3389/fonc.2024.1354837 (PMC11096596; doi:10.3389/fonc.2024.1354837)

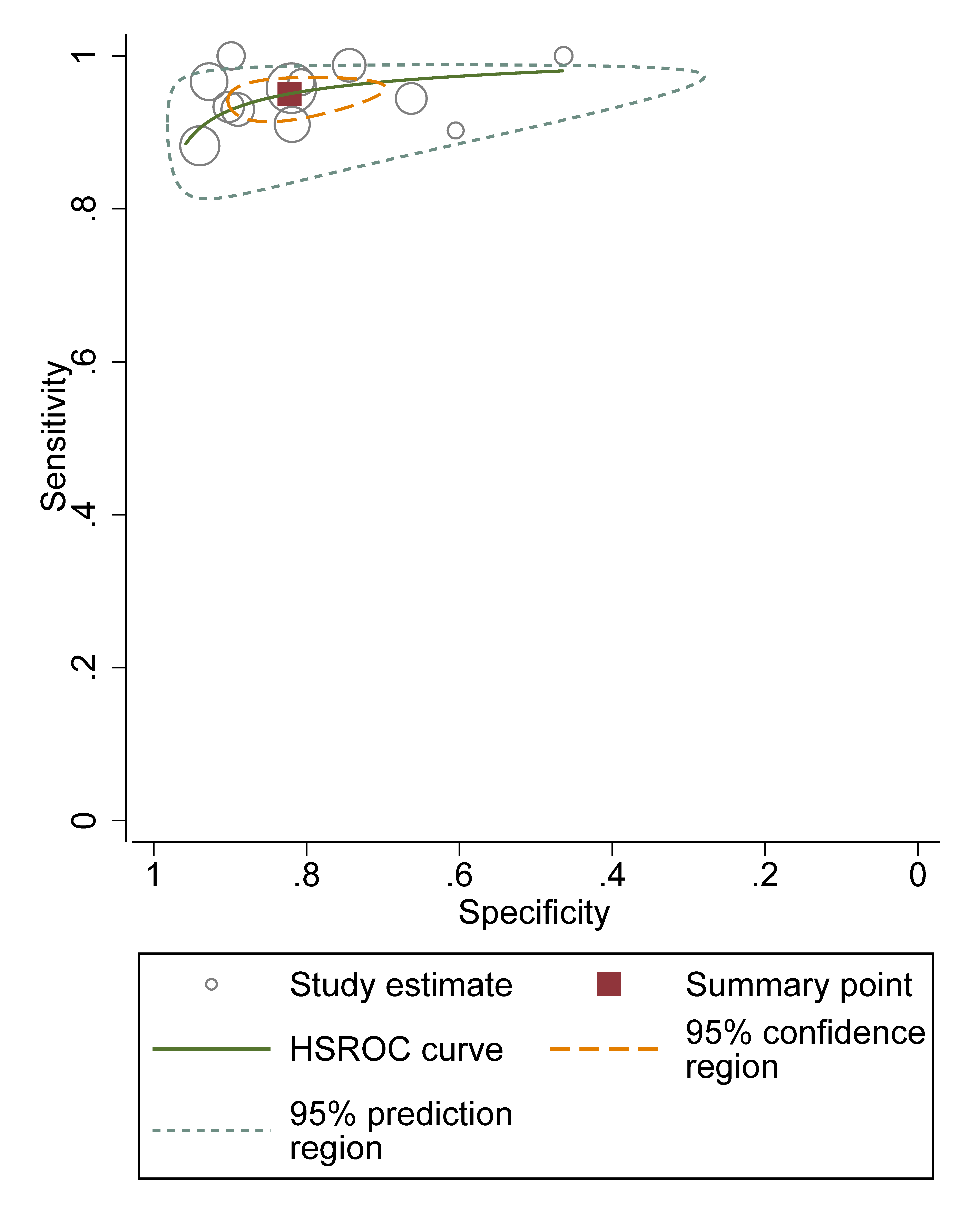

Supplement: Supplementary file 2 [file Image_1.tiff]

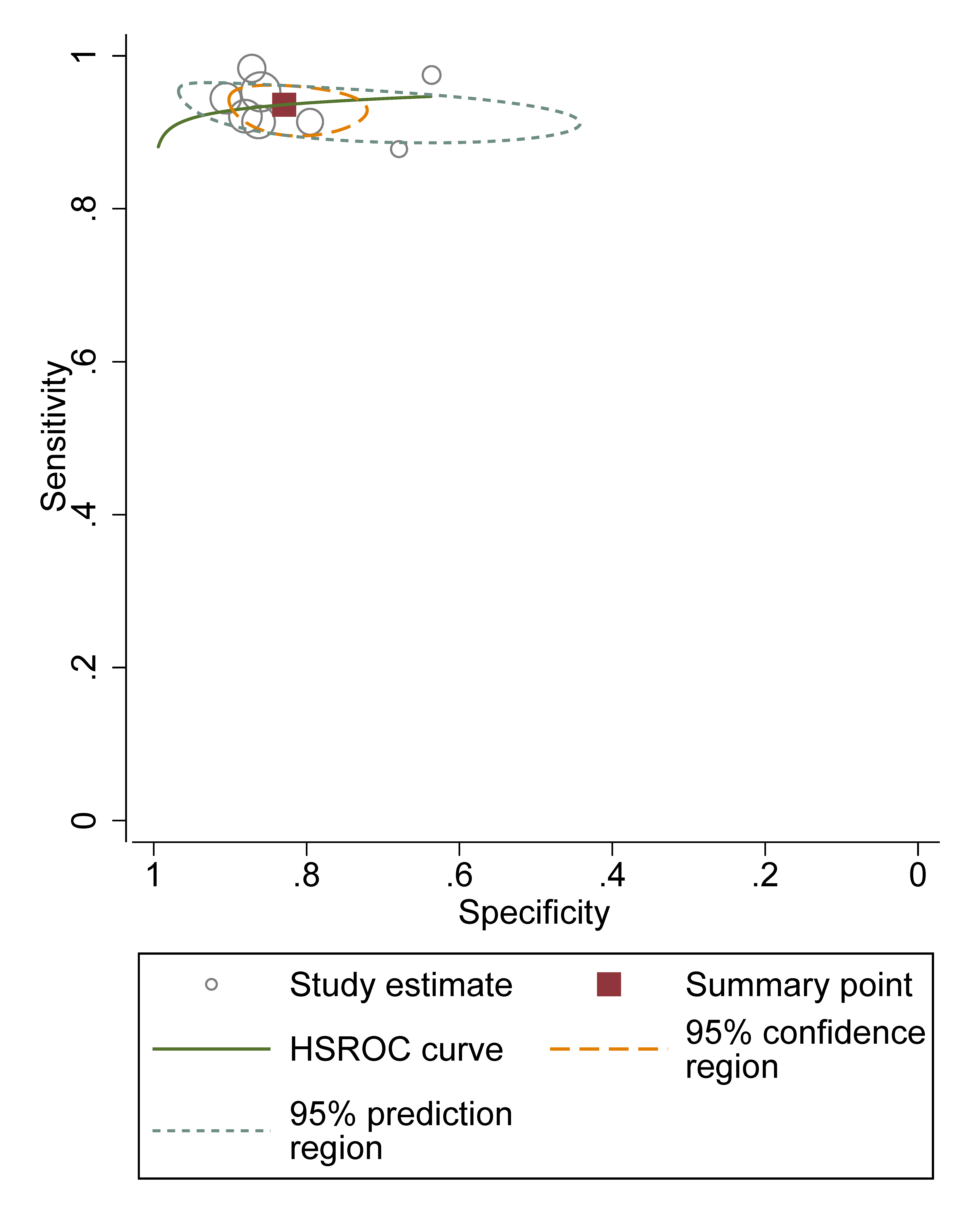

Supplement: Supplementary file 3 [file Image_2.tiff]

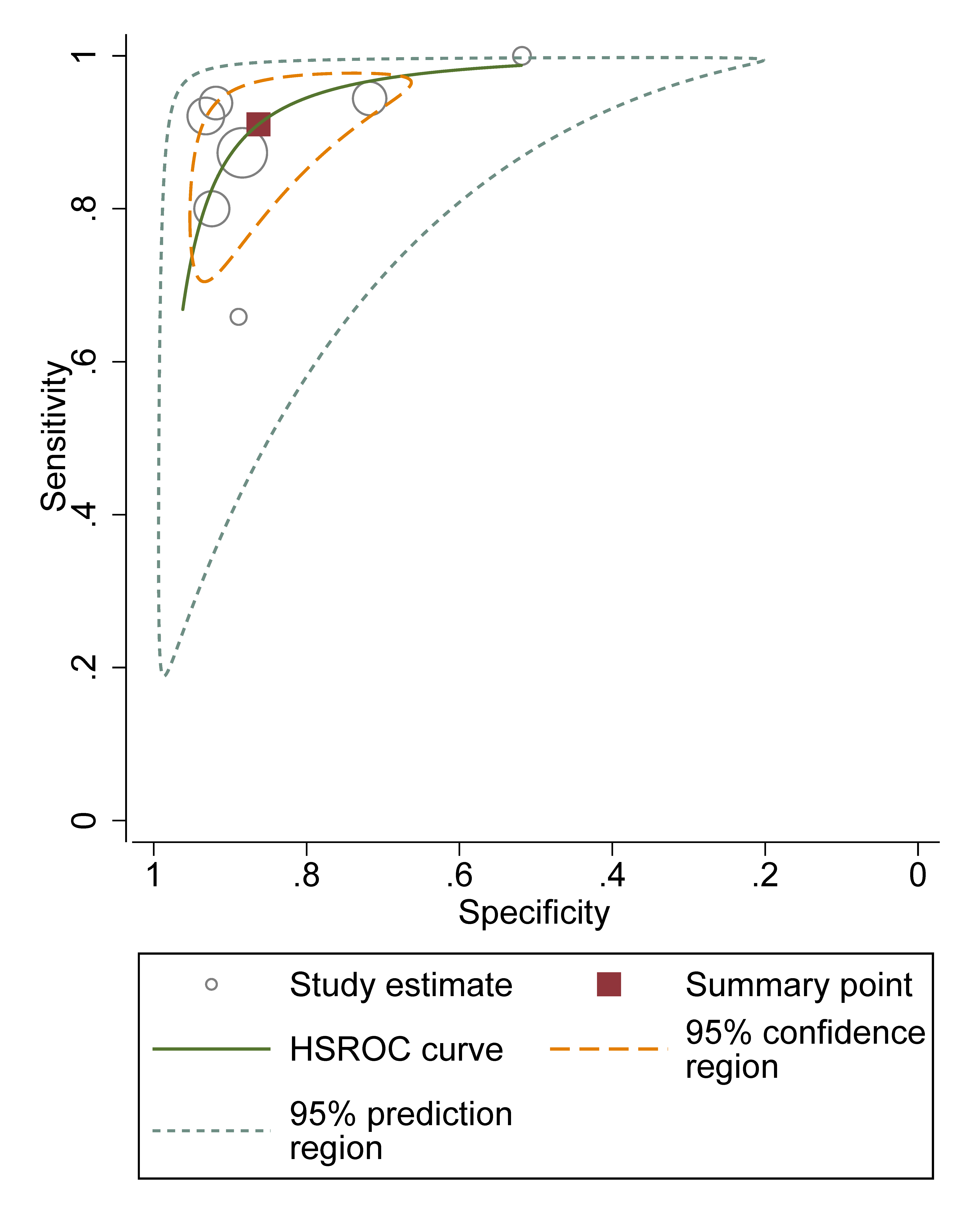

Supplement: Supplementary file 4 [file Image_3.tiff]
